# Relapse of tardive dyskinesia due to reduction in clozapine dose

**DOI:** 10.4103/0253-7613.56067

**Published:** 2009-08

**Authors:** Meena Shrivastava, Bhupendra Solanke, Ganesh Dakhale, Abhishek Somani, Pravir Waradkar

**Affiliations:** Department of Pharmacology, Indira Gandhi Government Medical College, Nagpur - 440 018, India; 1 Department of Psychiatry, Indira Gandhi Government Medical College, Nagpur - 440 018, India

**Keywords:** Clozapine, relapsed case, tardive diskinesia

## Abstract

Clozapine is a second-generation (atypical) antipsychotic agent, which has been proven efficient against the positive and negative symptoms of schizophrenia, with a low propensity to induce tardive dyskinesia (TD). Compared with typical antipsychotics, it has a greater affinity for dopamine D4 than D2 receptors and additional action on serotonin 5-HT_2A_ receptors. Due to its weak D_2_ blocking action, it produces few extra pyramidal side effects and TD is rare. TD is one of the muscular side effects of antipsychotic drugs, especially the older generation like haloperidol. TD does not occur until after many months or years of taking antipsychotic drugs. TD is primarily characterized by abnormal involuntary movements of the tongue, lips or jaw, as well as facial grimacing or extremities that develop in association with the use of antipsychotic medications. TD can be embarrassing to the affected patient in public. The movements disappear during sleep and women are at greater risk than men for developing TD.

## Introduction

It is established that clozapine and other second-generation antipsychotics cause less TD[1] and may also improve pre-existing TD.[2,3] Only few cases were reported having such complication. Hence, we wish to report a relapsed TD case due to reduction in clozapine dose from 200 mg to 150 mg.

## Case Report

A 46-year-old female patient of schizophrenia (DSM-IV) criteria came to OPD of psychiatry in Indira Gandhi Government Medical College and Hospital, Nagpur. This institute is also a regional center for reporting an adverse drug reaction for central India. The patient was suffering from schizophrenia since last 18 years and was on fluphenazine (depot) administered intramuscularly (25 mg) once in a month and without any evidence of movement disorder. Since last 6 months, she developed continuous chewing, lip licking, and pouting movements, and diagnosis of TD was done by a psychiatrist. She scored 7 on the Abnormal Involuntary Movement Scale. Her routine biochemical profile and hemogram was in normal limits. Then, fluphenazine administered intramuscularly was discontinued and the psychiatrist decided to start clozapine tablet in the dose of 50 mg twice daily, and after 1 week, the dose was increased to 100 mg twice daily. The total blood count was determined weekly. The patient returned after 2 weeks for follow-up with reduced symptoms of TD, no symptoms of schizophrenia, but complaining of excessive sedation because of clozapine.

Keeping in mind that sedation may be due to higher dose of clozapine, the psychiatrist decided to reduce the dose of clozapine to 150 mg (half tablet of 100 mg in the afternoon and 1 tablet at night). Again, 2 weeks later, the patient visited the OPD and exhibited increased symptoms of TD. A complete past history from relatives was taken regarding compliance of drugs. She was taking medication regularly and properly. The patient had decreased symptoms of TD after 10 days when the dose of clozapine again increased to 200 mg/day. The biochemical investigation and hemogram were normal. The patient is presently maintained on clozapine 200 mg/day without any re-emergence of TD since 3 months.

Till now, very few reports of clozapine-related TD are available in the existing literature. In one rare case, it was found that after 1 year of treatment with clozapine a patient developed TD.[4] Above-mentioned findings suggest that clozapine at the dose of 200 mg reduces symptoms of TD but when 150-mg dose of the same drug was given, symptoms of TD again re-emerged. A similar case of TD was reported with reduction in the dose of clozapine.[5] There is no proven effective treatment for TD. The available literature supports the use of clozapine in the management of TD.[1,2] The mechanism for TD is not well understood but the most commonly accepted explanation is super-sensitivity of post-synaptic dopaminergic receptors due to chronic dopaminergic blockade by antipsychotics. Older antipsychotics are more likely to cause this phenomenon, as they have stronger and irreversible dopamine blockade. Newer antipsychotic have serotonin- as well as dopamine-blocking property and slightly lower risk of TD.[6] All first-generation antipsychotic agents are associated with a risk of TD. Studies of newer antipsychotic agents suggest that TD liability is much lower with second-generation agents and clozapine is associated with substantial lower risk for the development of TD than other antipsychotic medication.[7] Although family and twin studies have elucidated the possible role of genetic factors, there is no significant association between single-nucleotide polymorphism and TD.[8,9]

In this patient, due to reduction in the dose of clozapine, the post-synaptic dopaminergic receptors may not be completely blocked and the increased amount of natural dopamine reaching these super-sensitized receptors leads to dysregulated movements giving rise to TD. However, the exact pathophysiological mechanisms are still not clear and more data are required to know the typical characteristics and risk factors associated with TD due to reduction in the clozapine dose.
